# Risk Factors for Severe Diarrhea with an Afatinib Treatment of Non-Small Cell Lung Cancer: A Pooled Analysis of Clinical Trials

**DOI:** 10.3390/cancers10100384

**Published:** 2018-10-15

**Authors:** Ashley M. Hopkins, Anh-Minh Nguyen, Christos S. Karapetis, Andrew Rowland, Michael J. Sorich

**Affiliations:** 1 College of Medicine and Public Health, Flinders University, Adelaide 5042, Australia; ash.hopkins@flinders.edu.au (A.M.H.); anhminh.nguyen@flinders.edu.au (A.-M.N.); c.karapetis@flinders.edu.au (C.S.K.); andrew.rowland@flinders.edu.au (A.R.); 2 Department of Medical Oncology, Flinders Medical Centre, Adelaide 5042, Australia

**Keywords:** EGFR inhibitor, diarrhea, toxicity, prediction model, adverse event

## Abstract

Afatinib is an effective therapy for metastatic non-small cell lung cancer (NSCLC) but it is associated with a relatively high incidence of severe diarrhea. The association between pre-treatment candidate predictors (age, sex, race, performance status, renal function, hemoglobin, and measures of body mass) and severe (grade ≥ 3) diarrhea was evaluated using logistic regression with pooled individual participant data from seven clinical studies. A risk score was developed based on the count of major risk factors. Overall, 184 of 1151 participants (16%) experienced severe diarrhea with use of afatinib. Body weight, body mass index, and body surface area all exhibited a prominent non-linear association where risk increased markedly at the lower range (*p* < 0.005). Low weight (<45 kg), female sex, and older age (≥60 years) were identified as major independent risk factors (*p* < 0.01). Each risk factor was associated with a two-fold increase in the odds of severe diarrhea, and this was consistent between individuals commenced on 40 mg or 50 mg afatinib. A simple risk score based on the count of these risk factors identifies individuals at lowest and highest risk (C-statistic of 0.65). Risk of severe diarrhea for individuals commenced on 40 mg afatinib ranged from 6% for individuals with no risk factors to 33% for individuals with all three risk factors.

## 1. Introduction

Small molecule epidermal growth factor receptor (EGFR) tyrosine kinase inhibitors are an effective treatment option for patients with non-small cell lung cancer (NSCLC) harboring a sensitizing EGFR mutation [1]. Use of afatinib, a second-generation EGFR inhibitor with favorable efficacy [2], has been tempered by toxicity concerns. Diarrhea is the most common severe adverse event associated with the use of afatinib and is observed at higher frequency compared to other EGFR inhibitors [3,4].

A series of relatively small studies have reported that the risk of diarrhea with afatinib may be associated with low body surface area (BSA) [5], low body mass index (BMI) [6], low hemoglobin [6], and female sex [7]. Furthermore, product information for afatinib (Giotrif^®^) highlights that female sex, low body weight, and renal impairment are associated with higher afatinib exposure (i.e., drug plasma concentration), and this may impact on the risk of toxicity [8,9]. This study aimed to evaluate putative pre-treatment predictors of severe diarrhea in a larger patient population by pooling data from clinical trials of afatinib for NSCLC, and to develop a simple tool that may be used in clinical practice to estimate an individual’s risk of severe diarrhea with afatinib.

## 2. Results

The pooled analysis set included 1151 participants using afatinib, of which 184 (16%) experienced severe diarrhea. Incidence of severe diarrhea differed between studies, ranging between 6% to 37%. The median time to grade 3–4 diarrhea was 18 days for individuals commenced on 40 mg afatinib and 16 days for individuals commenced on 50 mg afatinib. Overall and study-specific participant characteristics are summarized in Table 1 and Table S1, respectively.

**Figure 1:**
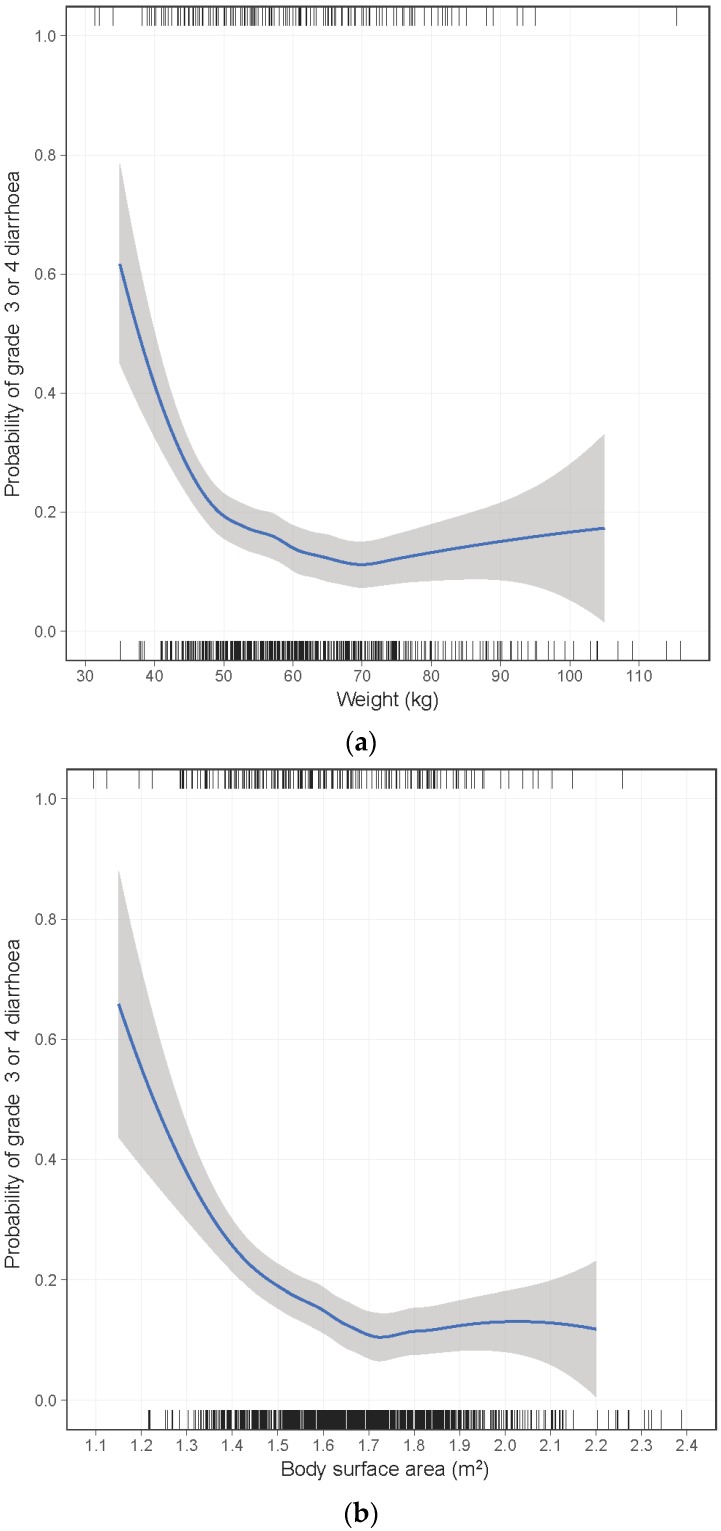

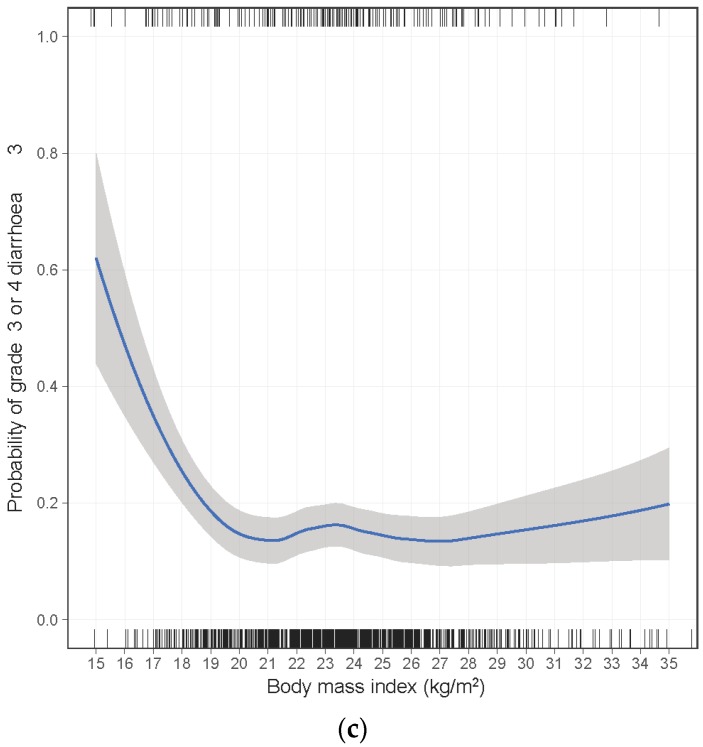
Loess locally weighted smoothed relationship between risk of grade ≥3 diarrhea and (**a**) body weight, (**b**) body surface area, and (**c**) body mass index.

A non-linear association was identified for body weight, BSA, and BMI with an increased risk of severe diarrhea primarily at the lower range (Figure 1). This association was present for both individuals commenced on 40 mg or 50 mg afatinib. Very few males were in the weight/BSA range for which risk was substantially increased (e.g., 3% of males vs. 22% of females had weight < 50 kg). To aid interpretation and reporting, continuous variables were categorized into four groups based on the visual inspection of the loess curves and established cut points. For weight, BSA, BMI, and eGFR categories were selected to evaluate risk at progressively lower values. For age and hemoglobin, no significant non-linearity was apparent. Consequently, hemoglobin was categorized into quartiles, and age was categorized into four similarly sized groups with cut-points falling at decades of age.

Univariate analysis identified older age, female sex, low body weight, low BSA, and low BMI was most strongly associated with an increased risk of severe diarrhea (*p* < 0.005, Table 2). Lower eGFR, non-Asian race, and lower hemoglobin had a trend toward increased risk of severe diarrhea (*p* < 0.1, Table 2). No significant heterogeneity in variable effect size was apparent between studies or between different starting doses (40 mg or 50 mg) of afatinib (*p* > 0.05). Low weight, low BSA, and low BMI were highly correlated and, as they provided similar predictive performance, for simplicity weight was selected for inclusion in the multivariable analysis.

Multivariable logistic regression analysis identified that older age, female sex, and low body weight were all significant independent predictors of severe diarrhea (*p* < 0.01, Table 2). A sensitivity analysis including adjustment for afatinib starting dose identified the same predictors (Table S2). A simplified risk score was subsequently constructed including these three major factors. For simplicity, age and weight were dichotomized using a single cut point identified from the results of the multivariable analysis. For age, a cut point of 60 years was selected on the basis that the effect size for the 60 to 69 years and ≥70 years age groups were similar and were substantially higher than that of the 50 to 59 years age group. A cut point of <45 kg body weight was selected on the basis that this would have an effect size similar to that of age and sex. As each risk factor had a similar independent effect size (approximately doubling the odds), which was consistent for both 40 mg and 50 mg starting afatinib doses, they were given equal weighting and the risk score was calculated as the simple count of the major risk factors (female sex, age ≥60 years, and body weight <45 kg).

The risk of severe diarrhea associated with each count of risk factors was evaluated separately for individuals commenced on 40 mg and 50 mg afatinib. The risk of severe diarrhea for individuals commenced on 40 mg afatinib ranged from 6% for individuals with no risk factors to 33% for individuals with all three risk factors (Table 3). For individuals commenced on 50 mg afatinib, the risk ranged from 8% to 43% (Table 3). The C-statistic (discrimination) was 0.65 for individuals commenced on 40 mg of afatinib and 0.66 for individuals commenced on 50 mg afatinib.

As a sensitivity analysis, a multivariable logistic regression prediction model was developed using the same three risk factors, but with age and weight modelled as continuous variables (Table S3). This model demonstrated similar discrimination performance (C-statistic of 0.65) and good calibration (Figure S1).

## 3. Discussion

This study, the largest to evaluate pre-treatment predictors of severe diarrhea with afatinib treatment for NSCLC, confirmed findings from smaller studies that female sex and very low body weight are independent risk factors, and further identified older age as an independent risk factor. A pragmatic method was developed that enabled the prediction of the risk of severe diarrhea based on the simple count of three risk factors.

It has been previously reported that individuals with female sex, low body weight, or reduced renal function have moderately higher afatinib plasma concentrations [9]. These prior analyses suggest that the higher risk associated with low body weight and female sex are likely mediated by increased exposure to afatinib. Notably, almost all individuals with low body weight (particularly those <45 kg) were female. eGFR did not demonstrate an independent association with risk of severe diarrhea once adjusted for weight, age and sex. Age was not previously identified as having a significant association with afatinib exposure [9], and was not significantly associated with risk of diarrhea in prior studies [5,6]. However, a univariate association between older age and dose reductions [10] and toxicity in general [11] has been reported.

This risk score can be used to identify individuals with relatively low risk and relative high risk of severe diarrhea with use of afatinib. For individuals with low risk, the risk score may help identify individuals for whom afatinib should be considered as a treatment option. For individuals identified as being at higher risk, the risk tool may enable planning for closer monitoring during the first weeks of therapy, consideration of a lower afatinib starting dose, or selection of an alternative EGFR inhibitor with lower risk of diarrhea. Although there appears to be a relationship between afatinib concentration and afatinib toxicity, there is currently only preliminary direct evidence to confirm that starting on an afatinib dose less than 40 mg will reduce the risk of toxicity [12]. Furthermore, the efficacy impact of commencing on a lower starting dose of afatinib is currently uncertain [12]. There are now multiple EGFR inhibitor options, including osimertinib [13], a third generation EGFR inhibitor. Further head-to-head studies are required to understand the optimal sequencing of EGFR inhibitors and the role of afatinib [14].

A potential limitation of this study is the representativeness of the clinical trial population. While the trials included use of afatinib across multiple lines of therapy, clinical trials may not represent the full range of individuals encountered in practice, e.g., patients with an ECOG (Eastern Cooperative Oncology Group) performance status of two or greater. It will be valuable to evaluate the performance of the count of these three risk factors in a large contemporary real-world cohort to ensure predictions are well calibrated. The performance of this risk score was not evaluated in an independent cohort. Although the risk of overfitting is relatively low due to the large sample size and the restricted set of candidate predictors evaluated, the validation of prediction performance in an independent cohort, preferably a large contemporary real-world cohort, is an important future direction.

Additionally, the analysis included studies with an afatinib starting dose of 50 mg, in addition to the contemporary use of 40 mg. The starting dose of afatinib was not formally included in the modelling of risk factors as there was minimal with-in study differences in the afatinib starting dose (only LUX-Lung 2 [15] included a mix of 40 mg and 50 mg commencing doses). Notably, no significant differences in the effect size of risk factors were evident between the two starting doses, thus supporting the use of all available data to identify the major risk factors. Final results of the model performance were presented separately for 40 mg and 50 mg commencing doses and similar discrimination performance was apparent between the different commencing doses. For individuals commenced on 40 mg afatinib, the risk of severe diarrhea was lower overall and was substantively increased only when at least two of the three risk factors were present.

Finally, age and weight were dichotomized in order to simplify prediction of risk. However, this likely resulted in some loss of information, particularly for age, which has a relatively linear association with risk. A cut point of 60 years was selected as it was the median age in the dataset, and on the basis of the multivariable analysis, risk was identified to be more prominent above this age. Although scored equivalently, an individual with an age of 40 years was likely to have a lower risk than an individual aged 55. With respect to weight, individuals of 45 to 50 kg were likely to have a modest increase in risk that was not accounted for. Nevertheless, the count of risk factors demonstrated comparable discrimination to the model including age and weight as continuous variables. This suggests that the simplifications were a reasonable trade-off, particularly if the limitations above are considered in clinical use.

## 4. Materials and Methods

### 4.1. Study Design and Patients

The study was a pooled secondary analysis of patients with advanced NSCLC across seven clinical trials sponsored by Boehringer Ingelheim: NCT00656136 (LUX-Lung 1) [16], NCT00525148 (LUX-Lung 2) [15], NCT00949650 (LUX-Lung 3) [17], NCT00711594 (LUX-Lung 4) [18], NCT01121393 (LUX-Lung 6) [19], NCT00796549 (BI-1200.40) [20], and NCT00730925 (BI-1200.41) [21]. Anonymized patient level data were accessed via clinicalstudydatarequest.com. Secondary analysis of anonymized participant-level trial data was approved by the Southern Adelaide Clinical Human Research Ethics Committee (SAC HREC EC00188, 4 December 2015). All patients treated with afatinib monotherapy were included in the analysis.

### 4.2. Predictor and Outcome Data

Severe diarrhea was defined as either grade 3 or 4 according to the National Cancer Institute Common Terminology Criteria for Adverse Events version 3.0. Patients were considered at risk of diarrhea while on afatinib therapy and up to 28 days after cessation.

Pre-treatment predictor variables were selected on the basis of clinical or biological plausibility and prior evidence of association [5,6,7,8,9]. Predictors evaluated included age, sex, race, body weight, BSA, BMI, estimated glomerular filtration rate (eGFR) [22], ECOG (Eastern Cooperative Oncology Group) performance status, and hemoglobin level. Afatinib starting dose was excluded from the analysis on the basis of insufficient within-study differences [15].

### 4.3. Statistical Analysis

The association between potential predictors and a serious diarrhea was evaluated using logistic regression adjusted for any between-study differences in diarrhea incidence. Association was reported as an odds ratio (OR) with a 95% confidence interval (CI). Due to minimal missing data, a complete case analysis was undertaken. Potential non-linear association of continuous variables was graphically displayed using loess smoothed curves [23]. To aid interpretability, continuous variables were categorized into four groups based on inspection of the loess curves, using standard cut-points where possible. Univariate analysis was initially undertaken to evaluate the crude association with each predictor. Between-study heterogeneity in the predictor effect was evaluated using statistical interaction with the study variable.

Multivariable logistic regression analysis included all variables from the univariate analysis, with the exception that due to the strong correlation between weight, BSA, and BMI, only one variable was included based on the univariate analysis. The results of the multivariable analysis were used to identify the major independent risk factors and reasonable cut points for any continuous risk factors. For simplicity, a single cut point was sought for each major risk factor identified, with the cut point preferably selected such that the major risk factors identified had similar effect size. The risk score was defined as the count of the major independent risk factors present.

To evaluate the performance of the risk score, individuals commenced on 40 mg afatinib were evaluated separately from individuals commenced on 50 mg afatinib. The observed risk of severe diarrhea associated with each risk score was estimated by the number of patient who experienced severe diarrhea divided by the total number patients. Discrimination performance of the risk score and the prediction model was evaluated using the area under the receiver operating characteristic curve (C-statistic) [24].

As a sensitivity analysis, a logistic regression model was developed using the multivariable fractional polynomial approach [25] and including the same major risk factors included in the risk score, but without categorization of continuous variables. Goodness-of-fit was visually assessed by plotting a loess calibration curve of predicted probabilities with observed risk [26]. All analyses were two-sided and undertaken using the R statistical environment version 3.3.0.

## 5. Conclusions

Low body weight, female sex, and older age are independent significant risk factors for severe diarrhea with use of afatinib for NSCLC. A simple count of risk factors can provide an estimate of an individual’s risk.

## Figures and Tables

**Table 1:** Summary of pre-treatment characteristics for study participants commencing afatinib.

Characteristic	Total No. 1151
Age (years)	60 (52–68)
Sex	
Male	443 (38%)
Female	708 (62%)
Race	
Asian	839 (73%)
White	299 (26%)
Other	13 (1%)
ECOG PS	
0	385 (33%)
1	726 (63%)
2	40 (3%)
Prior EGFR inhibitor	479 (42%)
Prior chemotherapy	595 (52%)
Afatinib starting dose	
40 mg	498 (43%)
50 mg	653 (57%)
Weight (kg)	
Median (IQR)	61 (53–70)
Missing	3 (<1%)
Body mass index (kg/m^2^)	
Median (IQR)	23 (21–26)
Missing	10 (1%)
Body surface area (m^2^)	
Median (IQR)	1.7 (1.5–1.8)
Missing	10 (1%)
eGFR (mL/min/1.73 m^2^)	
Median (IQR)	91 (76–109)
Missing	5 (<1%)
Hemoglobin (g/L)	
Median (IQR)	128 (117–138)
Missing	5 (<1%)

ECOG PS: Eastern Cooperative Oncology Group performance status; eGFR: Estimated glomerular filtration rate; IQR: interquartile range.

**Table 2:** Logistic regression analysis * of association between pre-treatment characteristics and grade ≥3 diarrhea with afatinib.

Baseline Characteristics	Univariate Analysis *				Multivariable Analysis *		
	Events/*N* (%)	OR	95% CI	*p*	OR	95% CI	*p*
Sex				<0.001			<0.001
Male	44/443 (10%)	1.00			1.00		
Female	140/708 (20%)	2.22	1.53–3.21		2.04	1.36–3.07	
Age (years)				<0.001			0.008
27–49	21/237 (9%)	1.00			1.00		
50–59	40/319 (13%)	1.44	0.82–2.53		1.38	0.78–2.47	
60–69	78/373 (21%)	2.57	1.52–4.33		2.32	1.35–4.00	
70–86	45/222 (20%)	2.24	1.27–3.96		1.97	1.08–3.61	
Race ^†^				0.080			0.079
Asian	121/839 (14%)	1.00			1.00		
Non-Asian	63/312 (20%)	1.45	0.96–2.20		1.49	0.96–2.32	
Weight (kg)				<0.001			0.003
≥50	138/977 (14%)	1.00			1.00		
45–49	22/102 (22%)	1.60	0.94–2.71		1.51	0.85–2.65	
40–44	16/56 (29%)	2.11	1.12–3.99		1.98	1.00–3.93	
<40	8/13 (62%)	8.81	2.72–28.5		7.93	2.32–27.1	
BMI (kg/m^2^)				0.002			
≥18.5	157/1063 (15%)	1.00					
17.0–18.4	13/58 (22%)	1.50	0.77–2.93				
16.0–16.9	7/14 (50%)	5.11	1.68–15.5				
<16.0	4/6 (67%)	10.2	1.66–62.5				
BSA (m^2^)				<0.001			
≥1.50	121/898 (13%)	1.00					
1.40–1.49	32/155 (21%)	1.65	1.05–2.60				
1.30–1.39	18/70 (26%)	2.13	1.17–3.86				
<1.30	9/17 (53%)	5.46	1.95–15.3				
eGFR (mL/min/1.73 m^2^)				0.066			0.248
≥90	74/591 (13%)	1.00			1.00		
60–89	92/478 (19%)	1.54	1.09–2.19		1.46	1.00–2.12	
45–59	14/64 (22%)	1.60	0.82–3.12		1.27	0.63–2.57	
<45	4/13 (31%)	2.17	0.63–7.46		1.72	0.48–6.16	
ECOG PS				0.460			0.306
0	71/385 (18%)	1.00			1.00		
1–2	113/766 (15%)	0.88	0.62–1.24		0.82	0.57–1.19	
Hemoglobin (g/L)				0.061			0.600
75–117	56/294 (19%)	1.00			1.00		
118–128	55/288 (19%)	1.07	0.70–1.64		1.03	0.66–1.61	
129–138	38/281 (14%)	0.69	0.43–1.09		0.76	0.47–1.23	
139–185	34/283 (12%)	0.63	0.39–1.02		0.91	0.54–1.53	

BMI: body mass index; BSA: body surface area; CI: confidence interval; ECOG PS: Eastern Cooperative Oncology Group performance status; eGFR: estimated glomerular filtration rate; N: number of patients; OR: odds ratio; * All logistic regression models were adjusted for any between-study differences in diarrhea incidence. ^†^ Limited to subset of studies (LUX-Lung 1, 2, and 3) that have within-study race differences.

**Table 3:** Risk of grade ≥3 diarrhea with afatinib treatment (40 mg or 50 mg starting dose) by count of risk factors ^1^.

Risk Factors ^1^	Events/Patients (%)	
	40 mg Afatinib	50 mg Afatinib
0	5/90 (6%)	9/119 (8%)
1	17/238 (7%)	51/312 (16%)
2	23/152 (15%)	64/198 (32%)
3	6/18 (33%)	9/21 (43%)

^1^ Female sex, age ≥60 years, and body weight <45 kg.

## Supplementary Materials

The following are available online at http://www.mdpi.com/2072-6694/10/10/384/s1. Table S1: Summary by study of pre-treatment participant characteristics. Table S2: Multivariable logistic regression analysis of association between pre-treatment characteristics and grade ≥3 diarrhea with afatinib, including adjustment for afatinib starting dose. Table S3: Coefficients of multivariable logistic regression model of severe diarrhea with afatinib. Figure S1: Calibration plot for the multivariable logistic regression model of severe diarrhea with afatinib.
