# Supplementary material for: Risk Factors for Severe Diarrhea with an Afatinib Treatment of Non-Small Cell Lung Cancer: A Pooled Analysis of Clinical Trials
Source: Cancers (Basel). 2018 Oct 15;10(10):384. doi: 10.3390/cancers10100384 (PMC6210178; doi:10.3390/cancers10100384)
Supplement: Supplementary file 1 [file cancers-10-00384-s001.pdf]

Supplementary Materials: Risk Factors for Severe Diarrhea with an Afatinib Treatment of Non-Small Cell Lung Cancer: A pooled Analysis of Clinical Trials.

Ashley M Hopkins, Anh-Minh Nguyen, Christos S Karapetis, Andrew Rowland and Michael J Sorich

Table S1. Summary by study of pre-treatment participant characteristics.

Baseline Characteristic	Total No. 1151	LUX-Lung 1 No. 390	LUX-Lung 2 No. 129	LUX-Lung 3 No. 229	LUX-Lung 4 No. 62	LUX-Lung 6 No. 239	BI-1200.40 No. 69	BI-1200.41 No. 33
Age (years)	60 (52–68)	58 (51–66)	61 (53–71)	62 (55–68)	65 (58–72)	58 (49–65)	66 (58–72)	66 (53–71)
Sex								
Male	443 (38%)	159 (41%)	54 (42%)	82 (36%)	14 (23%)	87 (36%)	35 (51%)	12 (36%)
Female	708 (62%)	231 (59%)	75 (58%)	147 (64%)	48 (77%)	152 (64%)	34 (49%)	21 (64%)
Race								
Asian	839 (73%)	261 (67%)	112 (87%)	165 (72%)	62 (100%)	239 (100%)	0 (0%)	0 (0%)
White	299 (26%)	121 (31%)	16 (12%)	61 (27%)	0 (0%)	0 (0%)	69 (100%)	32 (97%)
Other	13 (1%)	8 (2%)	1 (1%)	3 (1%)	0 (0%)	0 (0%)	0 (0%)	1 (3%)
ECOG performance score								
0	385 (33%)	92 (24%)	83 (64%)	92 (40%)	29 (47%)	48 (20%)	33 (48%)	8 (24%)
1	726 (63%)	268 (69%)	42 (33%)	137 (60%)	33 (53%)	191 (80%)	35 (51%)	20 (61%)
2	40 (3%)	30 (8%)	4 (3%)	0 (0%)	0 (0%)	0 (0%)	1 (1%)	5 (15%)
Prior EGFR inhibitor	479 (42%)	390 (100%)	0 (0%)	0 (0%)	62 (100%)	0 (0%)	0 (0%)	27 (82%)
Prior chemotherapy	595 (52%)	390 (100%)	71 (55%)	0 (0%)	62 (100%)	0 (0%)	28 (41%)	18 (55%)
Afatinib starting dose								
40 mg	498 (43%)	0 (0%)	30 (23%)	229 (100%)	0 (0%)	239 (100%)	0 (0%)	0 (0%)
50 mg	653 (57%)	390 (100%)	99 (77%)	0 (0%)	62 (100%)	0 (0%)	69 (100%)	33 (100%)
Weight (kg)								
Median (IQR)	61 (53–70)	62 (55–71)	62 (55–72)	60 (52–69)	51 (46–60)	60 (54–65)	67 (60–75)	73 (59–80)
Missing	3 (0%)	2 (1%)	0 (0%)	0 (0%)	0 (0%)	0 (0%)	0 (0%)	1 (3%)
Body mass index (kg/m^2)								
Median (IQR)	23 (21–26)	24 (21–26)	24 (22–26)	24 (21–26)	22 (20–24)	23 (21–24)	24 (23–26)	25 (23–28)
Missing	10 (1%)	6 (2%)	1 (1%)	0 (0%)	0 (0%)	0 (0%)	0 (0%)	3 (9%)
Body surface area (m^2)								
Median (IQR)	1.7 (1.5–1.8)	1.7 (1.5–1.8)	1.7 (1.5–1.8)	1.6 (1.5–1.8)	1.5 (1.4–1.6)	1.6 (1.5–1.7)	1.8 (1.6–1.9)	1.8 (1.6–1.9)
Missing	10 (1%)	6 (2%)	1 (1%)	0 (0%)	0 (0%)	0 (0%)	0 (0%)	3 (9%)
eGFR (ml/min/1.73 m^2)								
Median (IQR)	91 (76–109)	84 (70–101)	81 (67–93)	98 (86–115)	93 (80–111)	102 (84–122)	87 (74–99)	88 (75–98)
Missing	5 (0%)	4 (1%)	0 (0%)	0 (0%)	0 (0%)	0 (0%)	1 (1%)	0 (0%)
Hemoglobin (g/L)								
Median (IQR)	128 (117–138)	126 (115–136)	127 (111–140)	130 (119–140)	126 (118–135)	129 (121–141)	130 (119–137)	127 (117–139)
Missing	5 (0%)	4 (1%)	0 (0%)	0 (0%)	0 (0%)	1 (0%)	0 (0%)	0 (0%)

ECOG: Eastern Cooperative Oncology Group, eGFR: Estimated glomerular filtration rate, IQR: interquartile range.

Table S2. Multivariable logistic regression analysis of association between pre-treatment characteristics and grade ≥3 diarrhea with afatinib, including adjustment for afatinib starting dose.

Baseline Characteristic	Multivariable analysis *		
	OR	95% CI	*P*
Afatinib dose			<0.001
40 mg	1.00		
50 mg	2.16	1.47–3.17	
Sex			<0.001
Male	1.00		
Female	2.13	1.42–3.19	
Age (years)			0.001
27–49	1.00		
50–59	1.41	0.79–2.50	
60–69	2.51	1.47–4.30	
70–86	2.19	1.21–3.96	
Race †			0.098
Asian	1.00		
Non-Asian	1.37	0.94–2.00	
Weight (kg)			<0.001
≥50	1.00		
45–49	1.63	0.94–2.84	
40–44	2.27	1.17–4.42	
<40	9.54	2.89–31.5	
eGFR (ml/min/1.73 m^2)			0.308
≥90	1.00		
60–89	1.39	0.96–2.01	
45–59	1.23	0.62–2.46	
<45	1.97	0.55–7.06	
ECOG PS			0.090
0	1.00		
1+	0.74	0.52–1.05	
Hemoglobin (g/L)			0.505
75 to 117	1.00		
118 to 128	0.92	0.59–1.43	
129 to 138	0.70	0.44–1.13	
139 to 185	0.92	0.55–1.52	

BMI: body mass index; BSA: body surface area; CI: confidence interval, ECOG PS: Eastern Cooperative Oncology Group performance status, eGFR: estimated glomerular filtration rate, N: number of patients, OR: odds ratio.

Table S3. Coefficients of multivariable logistic regression model of severe diarrhea with afatinib.

Term	Coefficient	Standard Error
Intercept	−4.74	0.54
Weight $^{-2}$ (kg^{-2})	2233	683
Female sex	0.607	0.20
Age (years)	0.0329	0.0078

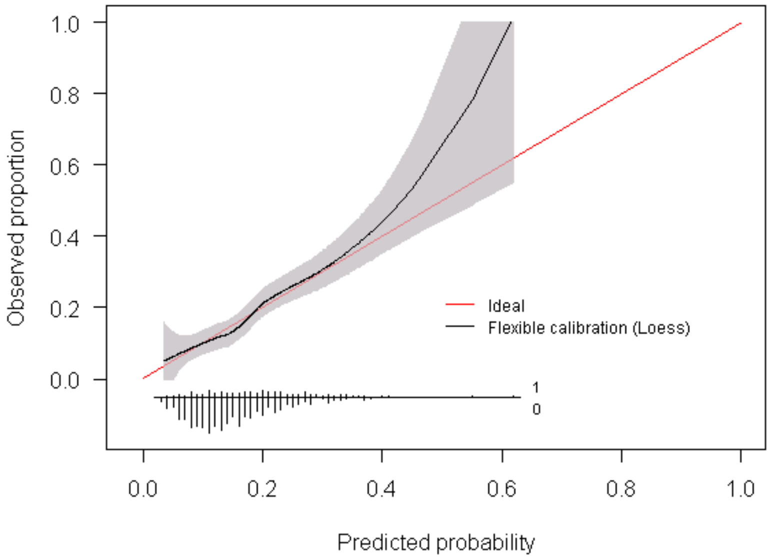

Figure S1. Calibration plot for the multivariable logistic regression model of severe diarrhea with afatinib.
